# Hypoglycemic encephalopathy mimicking acute ischemic stroke in clinical presentation and magnetic resonance imaging: a case report

**DOI:** 10.1186/s12880-019-0310-z

**Published:** 2019-01-24

**Authors:** Kai-I Chuang, Kevin Li-Chun Hsieh, Cheng-Yu Chen

**Affiliations:** 1 Department of Medical Imaging, Taipei Medical University Hospital, 252 Wu Hsing Street, Taipei, 110 Taiwan; 2 Research Center of Translational Imaging, College of Medicine, Taipei Medical University, 250 Wu Hsing Street, Taipei, 110 Taiwan; 3 Department of Radiology, School of Medicine, College of Medicine, Taipei Medical University, 250 Wu Hsing Street, Taipei, 110 Taiwan

**Keywords:** Hypoglycemic encephalopathy, Acute ischemic stroke, Diffusion-weighted imaging, Excitotoxic injury

## Abstract

**Background:**

The imaging findings of hypoglycemic encephalopathy can be considerably similar to those of ischemic infarction or toxic leukoencephalopathy. We demonstrated unusual magnetic resonance (MR) imaging features of hypoglycemic encephalopathy which can be confused with other pathology both on imaging and acute clinical presentation. The diffusion-weighted imaging (DWI) and apparent diffusion coefficients (ADC) map findings in our case further supports the hypothesis of hypoglycemia-induced “excitotoxic injury” of glial cells and myelin sheath that might protect neuron axons from intracellular edema and irreversible damage.

**Case presentation:**

A 72-year-old woman presented with poor appetite and was initially drowsy at home; the symptoms progressed to loss of consciousness accompanied by mild incontinence. The initial glucose level was 44 mg/dL, but no nausea, vomiting, fever, or cold sweating was reported. Physical examination after intravenous glucose supplementation revealed the absence of focal neurological signs, facial palsy, and tongue or eye deviations. The images obtained 24 h after symptoms onset revealed symmetrical hyperintensities on DWI (b-value: 1000) associated with hypointensities on ADC map along the corticospinal tract, from the levels of the cerebral peduncle and the posterior limbs of the internal capsule to the level of the corona radiata, which may mimic the imaging findings of acute ischemic infarction or amyotrophic lateral sclerosis. The patient received sliding-scale insulin therapy and rehabilitation, and she recovered consciousness without motor function deficits after 1 month. Moreover, repeat DWI and ADC map showed the complete disappearance of the lesions.

**Conclusions:**

In the phenomenon of excitotoxic injury, axons could be protected from intracellular edema and irreversible damage, which may explain the reversible clinical symptoms and imaging abnormality after controlling for blood glucose because of the preserved motor axon. The diagnosis of acute symptomatic hypoglycemic encephalopathy through clinical and imaging features can be challenging. It is crucial to differentiate it from ischemic encephalopathy since the management and clinical outcome are different.

**Electronic supplementary material:**

The online version of this article (10.1186/s12880-019-0310-z) contains supplementary material, which is available to authorized users.

## Background

The diagnosis of hypoglycemia-induced encephalopathy can be challenging clinically and can even be confounded by unusual neuroimaging findings. Hypoglycemia can be caused by a spectrum of medical conditions but is most commonly a result of underlying diabetes mellitus (DM). In patients with type 1 DM, hypoglycemia is caused by reduced sympathoadrenal responses, causing “hypoglycemia unawareness” [1]. However, in patients with type 2 DM, hypoglycemia is largely due to hypoglycemic agent overdose. The neurologic symptoms of hypoglycemia vary and include memory loss, motor function deficits, a persistent vegetative state and deep coma, or even death [2]. Furthermore, the imaging findings of hypoglycemic encephalopathy can be considerably similar to those of ischemic infarction or toxic leukoencephalopathy due to the common findings of water diffusion restriction on diffusion-weighted images (DWI) and apparent diffusion coefficients (ADC) map of magnetic resonance (MR) imaging [3]. Herein, we present a case of hypoglycemic encephalopathy with unusual DWI and ADC map findings at diagnosis and after glucose supplementation.

## Case presentation

**Fig. 1:**
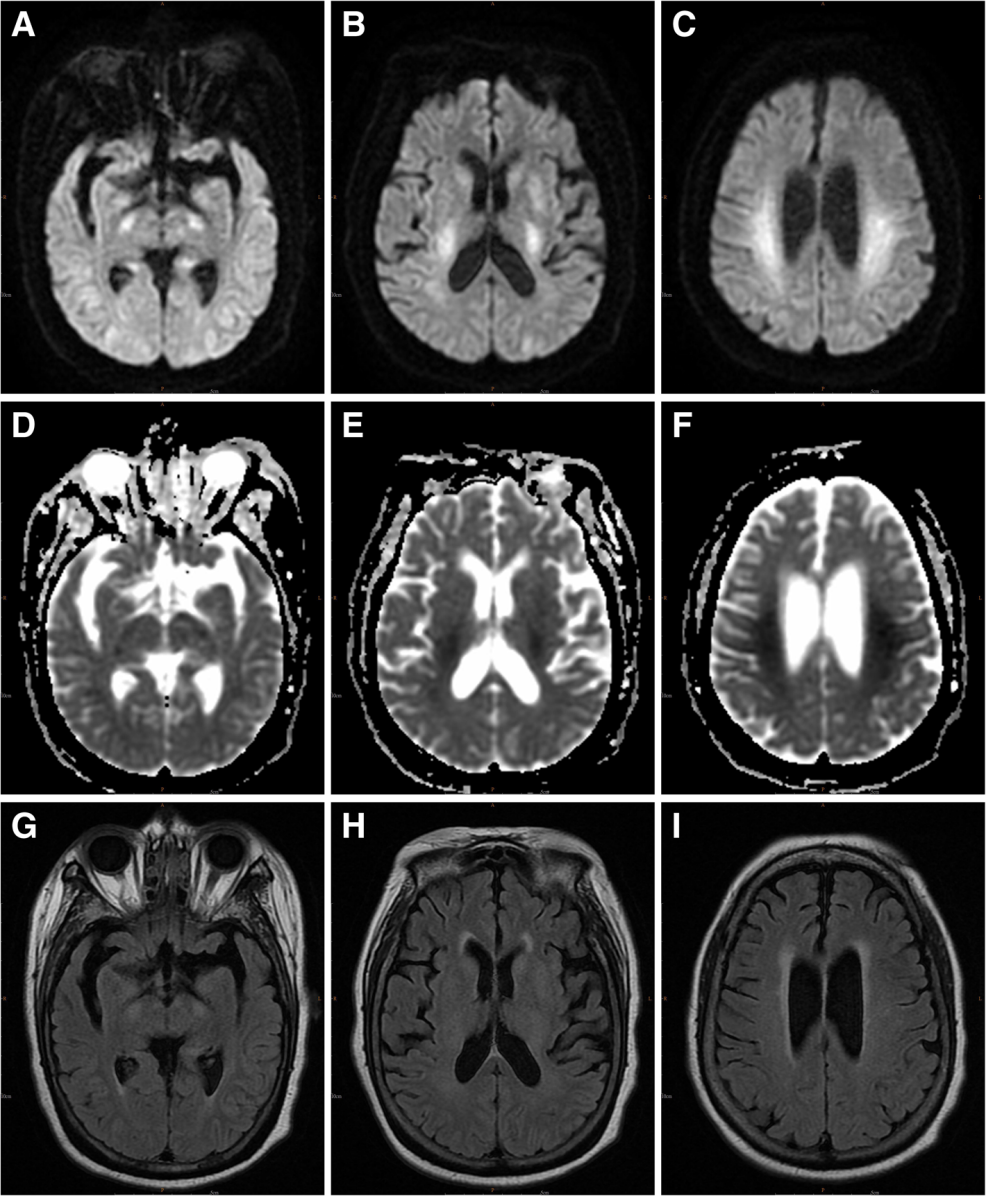
Initial axial magnetic resonance imaging conducted at the acute stage of hypoglycemic encephalopathy. Axial diffusion-weighted imaging (**a**, **b**, **c**), apparent diffusion coefficients map (**d**, **e**, **f**), and T2-FLAIR imaging (**g**, **h**, **i**) revealed only water diffusion restriction along the corticospinal tract, from the levels of the cerebral peduncle (**a**, **d**, **g**) and the posterior limbs of the internal capsule (**b**, **e**, **h**) to the level of the corona radiata (**c**, **f**, **i**), which occasionally mimic the imaging findings of acute ischemic stroke

**Fig. 2:**
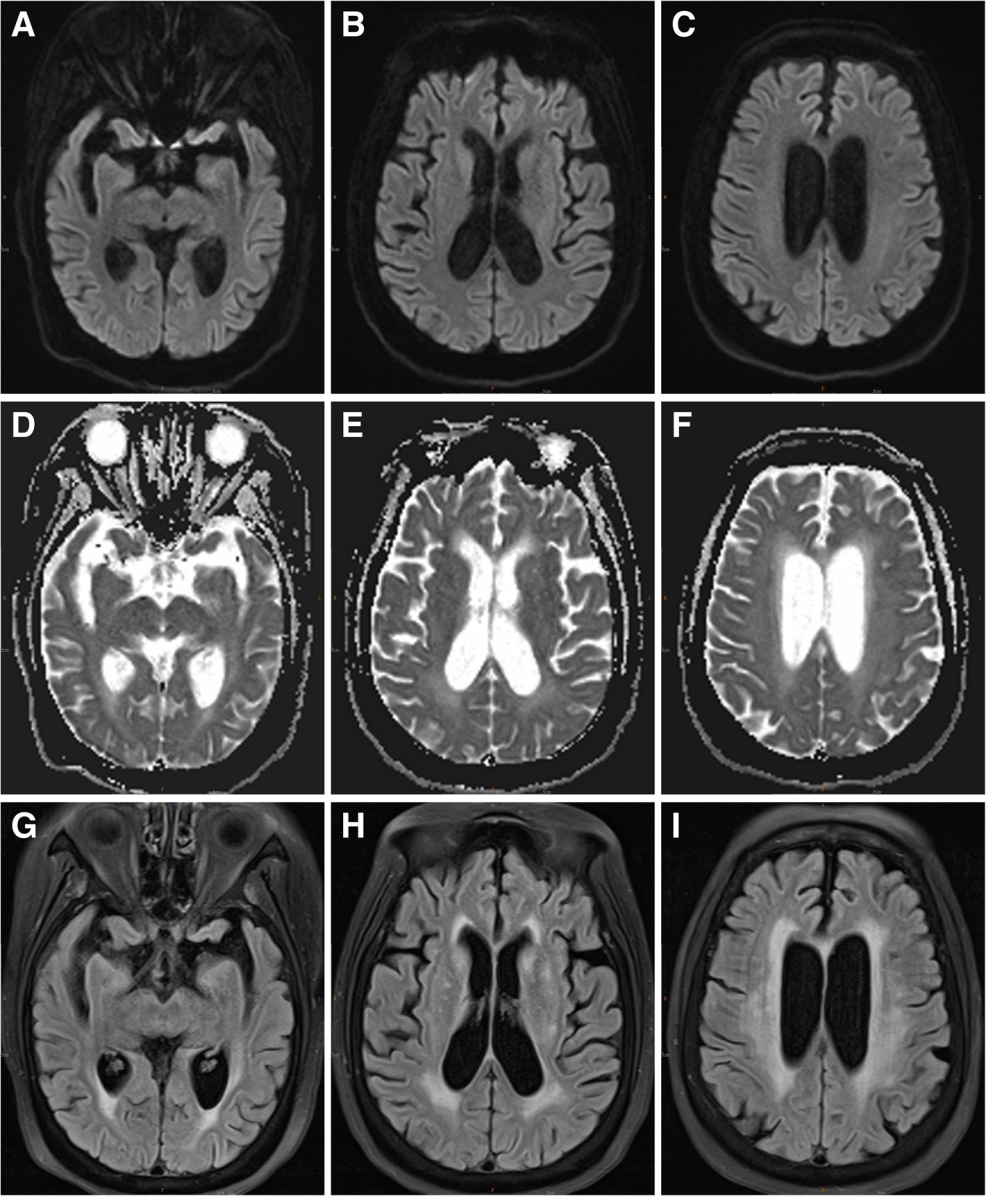
Repeat axial magnetic resonance imaging conducted 1 month after the patient received sliding-scale insulin therapy and rehabilitation. Axial diffusion-weighted imaging (**a**, **b**, **c**), apparent diffusion coefficients map (**d**, **e**, **f**), and T2-FLAIR imaging (**g**, **h**, **i**) revealed disappearance of the lesions along the corticospinal tract, from the levels of the cerebral peduncle (**a**, **d**, **g**) and the posterior limbs of the internal capsule (**b**, **e**, **h**) to the level of the corona radiata (**c**, **f**, **i**), possibly owing to the preservation of motor axons by the presence of the intramyelinic clefts

A 72-year-old woman presented with poor appetite and was initially drowsy at home; the symptoms progressed to loss of consciousness accompanied by mild incontinence. Therefore, the patient was admitted to the emergency department with an initial glucose level of 44 mg/dL, and no nausea, vomiting, fever, or cold sweating was reported. After intravenous glucose supplementation, she partially recovered consciousness (Glasgow Coma Scale [GCS]: E2V2M3), and her serum glucose level increased to 242 mg/dL. Physical examination revealed the absence of focal neurological signs, facial palsy, and tongue or eye deviations; however, mildly increased deep tendon reflexes were noted at the bilateral lower limbs. The images obtained 24 h after symptoms onset revealed symmetrical hyperintensities on DWI (b-value: 1000) associated with hypointensities on ADC map along the corticospinal tract, from the levels of the cerebral peduncle and the posterior limbs of the internal capsule to the level of the corona radiata, but there was no abnormal signal on T2-fluid attenuated inversion recovery (FLAIR) images (Fig. 1), which may mimic the imaging findings of acute ischemic infarction or amyotrophic lateral sclerosis. In-hospital electroencephalography indicated only generalized cortical dysfunction without evidence of focal seizure. The patient received sliding-scale insulin therapy and rehabilitation and recovered consciousness. A comprehensive neurological examination performed 1 month since the initial event of loss of consciousness revealed total recovery without motor function deficits. Moreover, repeat DWI (b-value: 1000) and ADC map showed the complete disappearance of the lesions (Fig. 2, Additional file 1).

## Discussion and conclusions

Hypoglycemic encephalopathy can cause reversible cytotoxic edema mostly in the cerebral cortices and deep-seated nuclei including the globus pallidus and thalami, whereas white matter involvement is observed only in the later stage [1–3]. However, the correlation between the degree of hypoglycemic levels and brain edema remains unclear. Our patient showed moderate hypoglycemia (44 mg/dL) but extensive white mater edema that involved the motor tract, which can mimic conditions such as acute ischemic infarction, amyotrophic lateral sclerosis, heroin intoxication, or other toxic leukoencephalopathy [4, 5]. Grey matter including the cortical mantle and globus pallidus was relatively spared.

In approximately 20% of acute hypoglycemia cases, the imaging features on DWI are similar to those of ischemic stroke [1–3]. Both glucose deprivation and ischemia lead to ionic pumping failure in the cell membrane [6, 7]. Nevertheless, hypoglycemia causes an increase in the extracellular glutamate level of motor neurons, which leads to the overexcitation of non-N-methyl-D-aspartate receptors, resulting in edema (fluid accumulation) in the surrounding myelin sheaths and glial cells. Hence, motor neurons are separated from the myelin layers by the presence of intramyelinic clefts and edema [2, 8, 9]. In this phenomenon of excitotoxic injury, axons could be protected from intracellular edema and irreversible damage [2, 8, 9], which may explain the reversible clinical symptoms and imaging abnormality after controlling for blood glucose.

The disease outcome was affected by several factors. In addition to the severity and duration of the hypoglycemia, the distribution of water diffusion restriction seen on DWI and ADC map, and recovery or not on follow-up MR images may predict the clinical outcome [2, 3, 10]. In general, lesions located anywhere along the corticospinal tracts without cerebral cortex involvement, and regression of the lesions on follow-up images are reported to be associated with a favorable clinical outcome [2, 10]. In the presented case, the patient had a favorable outcome with recovery of the consciousness and partial disappearance of the lesions on follow-up MR images after 1 month.

This study is constrained by several limitations. First, the follow-up MR study was performed at 1 month. The DWI and ADC map abnormality in ischemic stroke would also disappear at this time point [1]. Therefore, it is difficult to claim that the evolution of DWI and ADC map abnormality is different between hypoglycemia and ischemia without earlier MR study. Second, the presentation was a unique case. Further prospective studies with larger sample can validate the predictive value of DWI on hypoglycemic encephalopathy.

In conclusion, the diagnosis of acute symptomatic hypoglycemic encephalopathy through clinical and imaging features can be challenging. It is crucial to differentiate it from ischemic infarction since the management and clinical outcome are different.

## Additional file

Additional file 1: Medical History Timeline. (DOCX 19 kb)

## Abbreviations

ADC: Apparent diffusion coefficients
DM: Diabetes mellitus
DWI: Diffusion-weighted images
FLAIR: Fluid attenuated inversion recovery
GCS: Glasgow coma scale
MR: Magnetic resonance
