# Supplementary material for: Hypoglycemic encephalopathy mimicking acute ischemic stroke in clinical presentation and magnetic resonance imaging: a case report
Source: BMC Med Imaging. 2019 Jan 24;19:11. doi: 10.1186/s12880-019-0310-z (PMC6344988; doi:10.1186/s12880-019-0310-z)
Supplement: Supplementary file 1 — Medical History Timeline. (DOCX 19 kb) [file 12880_2019_310_MOESM1_ESM.docx]

Medical History Timeline

| Date | Relevant Past Medical History and Interventions | | |
| --- | --- | --- | --- |
| 2016-01 | A 72-year-old woman presented with poor appetite and was initially drowsy at home | | |
| Date | Summaries from initial and Follow-up Visits | Diagnostic Testing (including dates) | Interventions |
| 2016-01-31 | The symptoms progressed to loss of consciousness accompanied by mild incontinence. The initial glucose level was 44 mg/dL, but no nausea, vomiting, fever, or cold sweating was reported. | Brain MRI | Intravenous glucose supplementation, sliding-scale insulin therapy, and rehabilitation |
| 2016-03-02 | The patient recovered consciousness without any motor function deficits | Physical examination, brain MRI | rehabilitation |
